# Simultaneous Vascular Reconstruction and Cervical Anastomosis in McKeown Esophagectomy

**DOI:** 10.3389/fsurg.2021.646811

**Published:** 2021-04-08

**Authors:** Lei Chen, Jiaheng Zhang, Donglai Chen, Yonghua Sang, Wentao Yang

**Affiliations:** ^1^Department of Thoracic Surgery, The Second Affiliated Hospital of Soochow University, Suzhou, China; ^2^Department of Thoracic Surgery, School of Medicine, Shanghai Pulmonary Hospital, Tongji University, Shanghai, China

**Keywords:** right gastroepiploic artery, right gastroepiploic vein, vascular reconstruction, gastric conduit, McKeown esophagectomy

## Abstract

A stomach was considered ineligible to be an ideal conduit conventionally if its right gastroepiploic artery (RGEA) were injured. However, both sufficient blood flow and good venous return are crucial to the success of reconstruction. And there lacks robust evidence regarding the surgical techniques of reconstructing RGEA and right gastroepiploic vein (RGEV) and performing cervical anastomosis with gastric conduit simultaneously. Herein, we summarized the key surgical techniques for simultaneous vascular reconstruction and gastric conduit anastomosis in McKeown esophagectomy.

## Introduction

**Figure 1:**
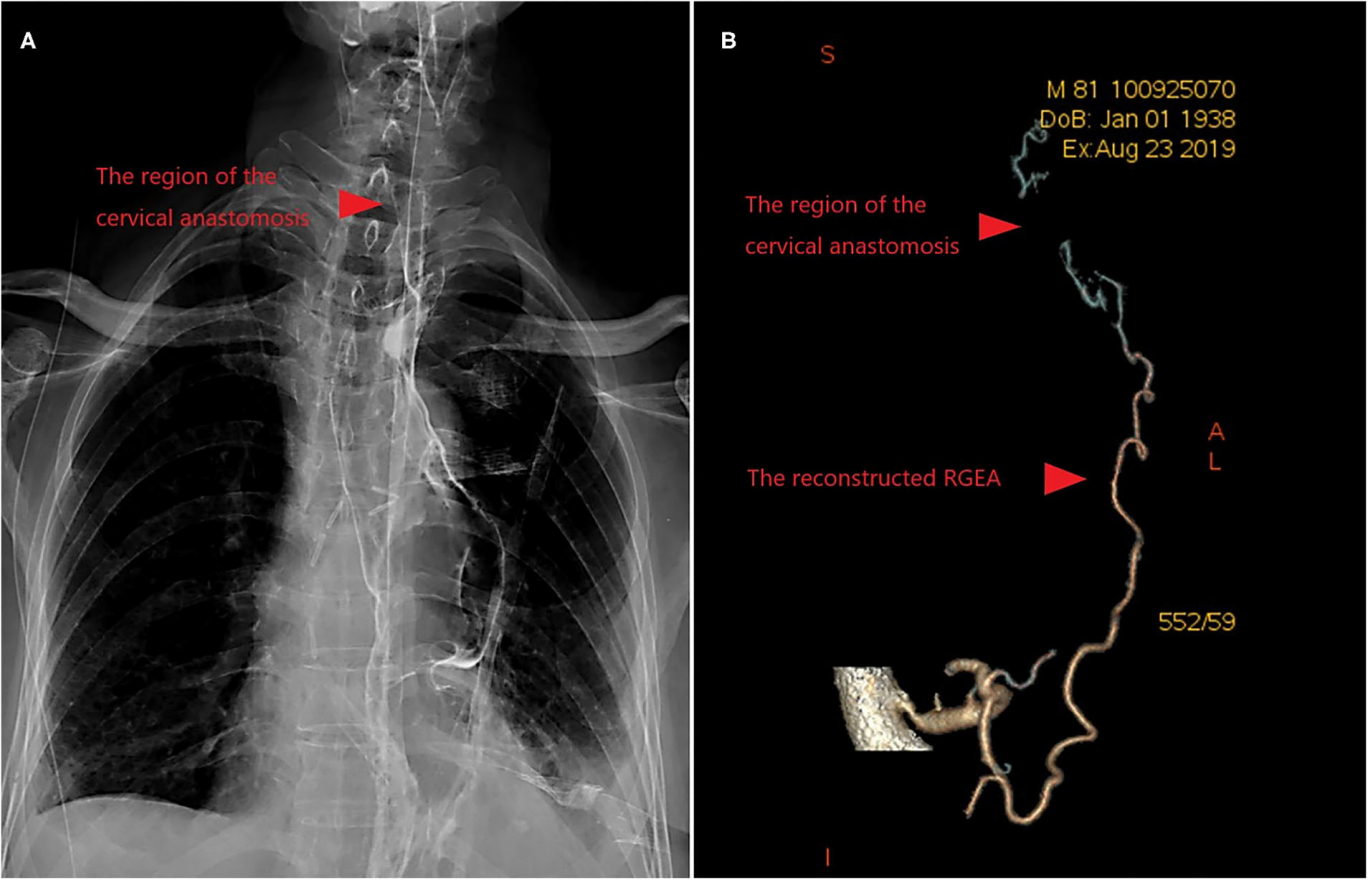
**(A)** Upper Gastrointestinal Contrast showed that there was no anastomosis leakage after 2 weeks of conservative treatment. **(B)** The postoperative assessment of blood supply was good revealed by Contrast-Enhanced CT.

McKeown esophagectomy is the primary surgical procedure for esophageal malignancies. As RGEA is the primary source of blood supply of the gastric conduit (1), the unavailability of RGEA disallows the stomach as an ideal substitute for esophagus. Instead, surgeons have to replace the esophagus with colon or jejunum (2, 3). In addition to the intactness of RGEA, unimpeded venous return in RGEV should be highlighted as well. Notably, in recent years, there have been rare reports on the exploration of intraoperative reconstruction of RGEA and RGEV. Moreover, the key surgical techniques during the vascular reconstruction and cervical anastomosis with gastric conduit has not been fully revealed in McKeown esophagectomy. In the past decade, a total of 843 patients received esophagectomy in our department, among whom 3 (0.36%) underwent vascular reconstruction in McKeown esophagectomy. All the three patients had good prognosis. One elderly patient with emphysema suffered from mild anastomotic leakage and respiratory failure after operation. The anastomotic leakage was cured after 2 weeks of conservative treatment (Figure 1A). In the present study, we summarized the surgical procedures for simultaneous reconstruction of RGEA and RGEV as well as gastric conduit anastomosis in McKeown esophagectomy based on our previous practice.

## Surgical Techniques

### Vascular Reconstruction and Assessment of Blood Flow

A midline incision was made in the epigastrium to ensure adequate relaxation of the gastric tissues and immediate exploration of the injured vessels. If the vascular deficit is small, the soft tissues around the vascular stumps should be fully dissociated, and then the tension of the vascular stumps should be accurately assessed. Once acceptable tension was identified at the vascular stumps, the injured vessels could be reconstructed via direct anastomosis promptly.

**Figure 2:**
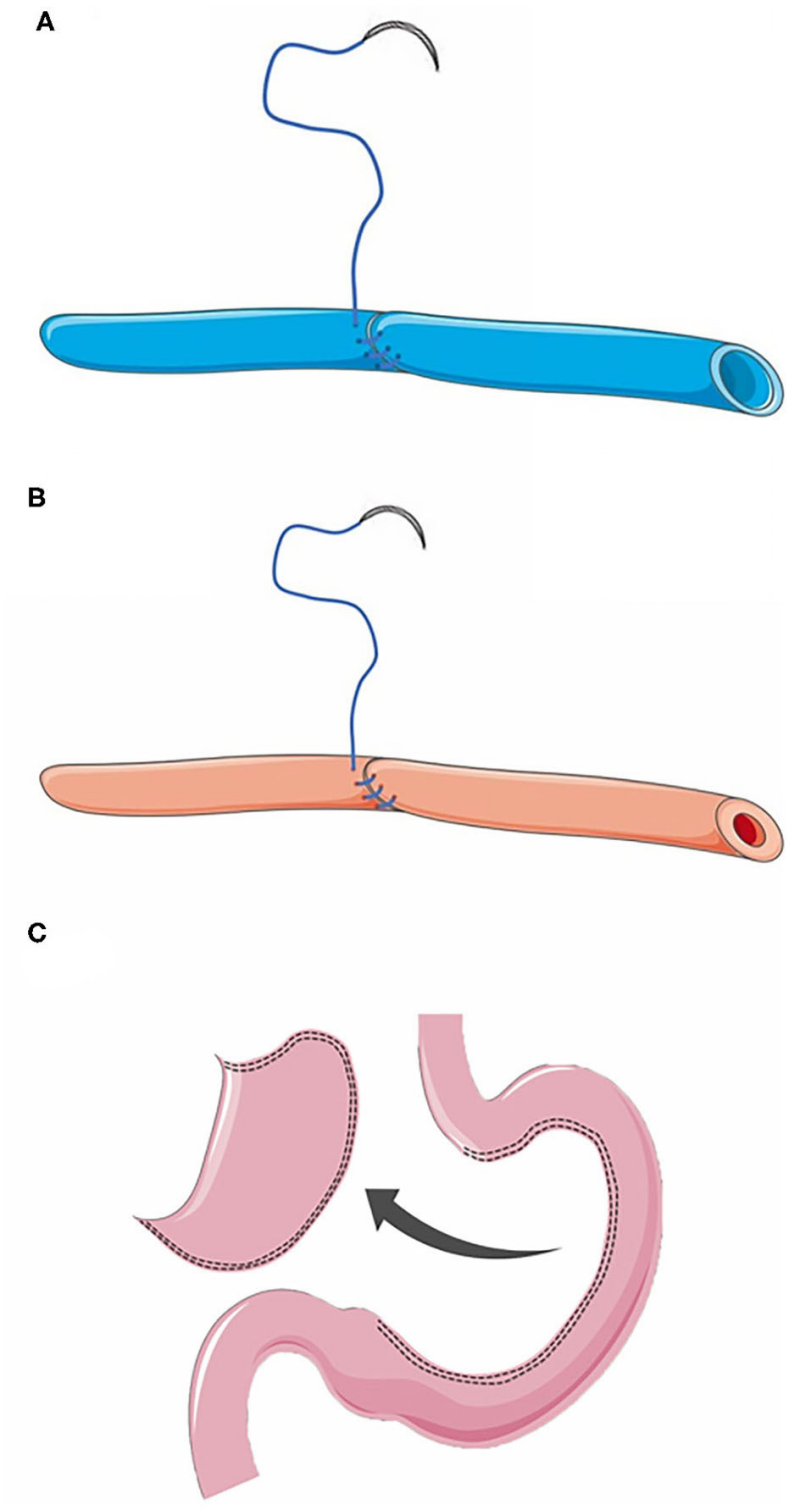
**(A)** Anastomosis of the right gastroepiploic vein. **(B)** Anastomosis of the right gastroepiploic artery. **(C)** Preparation of a gastric conduit without resection of gastric fundus during the gastric conduit construction until cervical anastomosis was completed.

**Figure 3:**
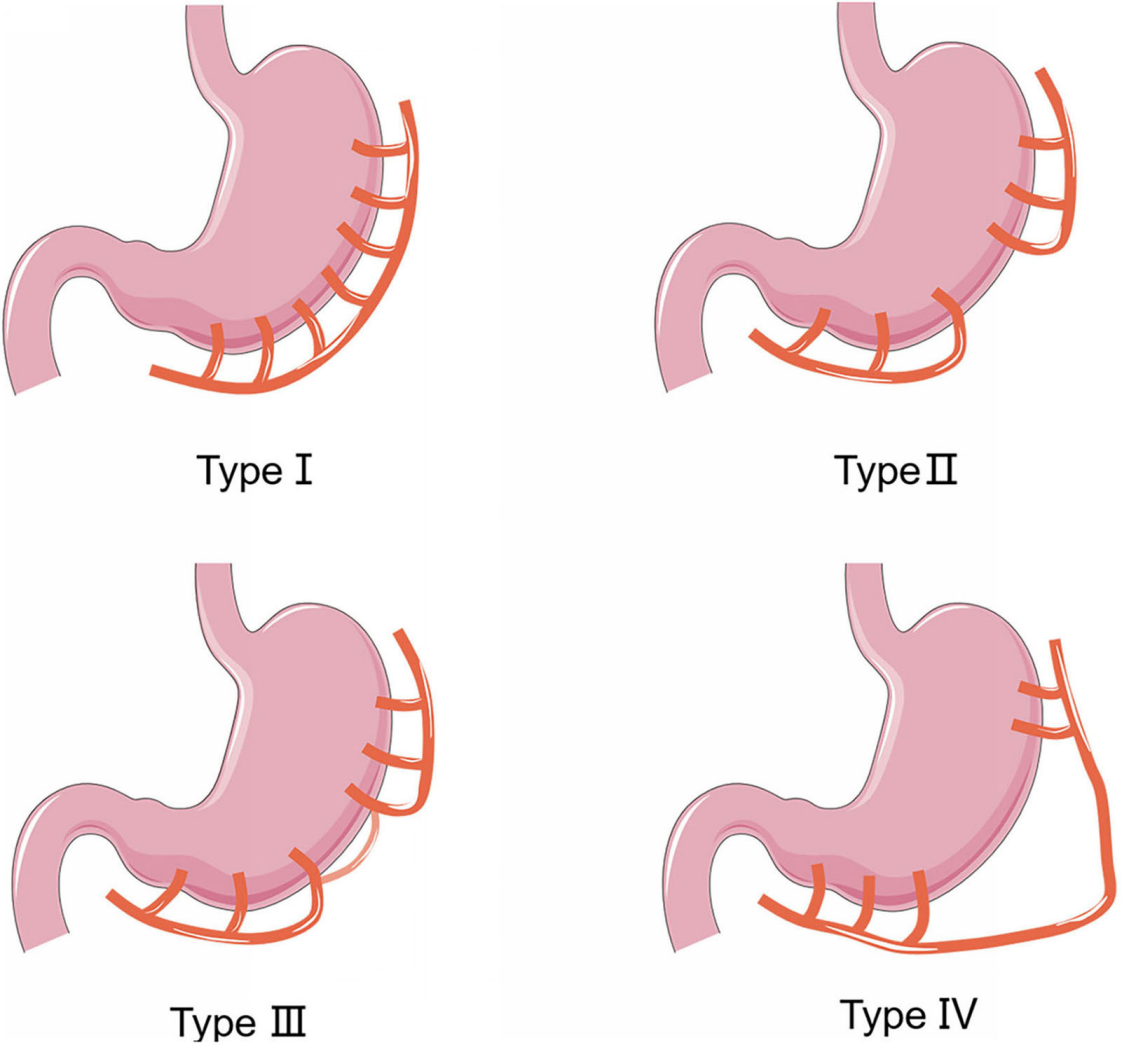
Four types of anatomic variations of the right gastroepiploic artery.

The principles for vascular anastomosis: (1) Both arteries and veins should be anastomosed by vein-first surgical technique; (2) The vessel stumps should be trimmed into an oblique section, after which continuous suture could be performed from the posterior wall of the vessel stumps using a 7-0 polypropylene thread (Figures 2A,B); (3) No additional suture on the stumps unless obvious bleeding after reperfusion; (4) Sufficient drainage in the abdominal cavity.

The blood flow after anastomosis could be accurately assessed intraoperatively with coronary blood flow measuring instrument. Once poor blood flow was found, the vascular reconstruction must be abandoned. Postoperative assessment of blood supply was performed by contrast-enhanced CT (Figure 1B) or angiography.

### Maintenance of Blood Sufficiency Intra- and Postoperatively

Once the blood perfusion of the anastomosed vessels was disturbed, it will greatly increase the risk of anastomotic leakage. Surgeons should pay attention to preventing thrombosis. Intra-operative anticoagulation therapy should be implemented with diluted heparin (5000 U/single dose), post-operative anticoagulation therapy with low molecular weight heparin (4000 AxaIU/qd), and followed by aspirin (100 mg/qd) for 1 year (Supplementary Table 1).

During the early postoperative period, sufficient blood capacity should be maintained to achieve appropriate blood pressure. Drugs that constrict peripheral blood vessels should be used with caution, so as to ensure adequate perfusion to the reconstructed RGEA.

### Ensure Adequate Anastomotic Tension of the Vessels and the Gastric Conduit

Minimized tension of vascular anastomosis and gastric conduit anastomosis as follows may be effective to avoid postoperative complications, such as esophageal anastomotic fistula and vascular anastomosis hemorrhage.

Before anastomosis, the tissues around the vascular stump should be fully freed to reduce the tension of the vascular stumps. To extend the length of the gastric conduit and to reduce the tension of esophageal anastomosis, the fundus of stomach should not be clipped during the gastric conduit construction until cervical anastomosis was completed (Figure 2C), while the adhesions surrounding the gastric conduit should be dissected sufficiently and cautiously.

After the operation, the gastric tube was placed in the lowest position of the gastric conduit to avoid gastric fluid retention. Enteral nutrition support via jejunostomy was recommended to avoid the physical stimulates from the nutrient tube. Those strategies can eliminate excessive internal tension in the gastric conduit, especially in the pylorus, so as to avoid the local expansion of gastric conduit which may increase the tension of vascular anastomosis.

## Discussion

Hitherto, there have been no convincing reports that revealed the feasibility and safety to reconstruct RGEA and RGEV and to perform cervical anastomosis using gastric conduit simultaneously. Given that a previous study introduced the cases on whom RGEA reconstruction was performed in Ivor-Lewis esophagectomy (1), the present study provided a novel perspective for thoracic surgeons who might intraoperatively injured the RGEA in the McKeown esophagectomy. In the present study, 2 patients received reconstruction of RGEA and RGEV in the vein-first order, as both the RGEA and RGEV were injury during the operation. While, another patient received reconstruction of RGEA, as only the RGEA was injury during the operation. The vascular reconstruction and cervical anastomosis using gastric conduit were performed simultaneously in all patients without severe postoperative complication. And no patient died in 6 months after surgery.

### Previous Treatment for RGEA and RGEV Disuse

In addition to cancerous involvement, anatomical variations (Figure 3) or previous damages on the vessel, the main causes of injuries on the RGEA and RGEV are severe tissue adhesion (2, 3). Once the vessels were severed during the operation, surgeons used to perform gastrectomy and esophageal reconstruction with a long colon or jejunum segment, which may cause greater risk of complications (2). Therefore, it is of great clinical significance to ensure effective vascular reconstruction.

### The Key Surgical Techniques

Based on the literature review and clinical experience, we would like to underscore 2 points of the surgical techniques in McKeown esophagectomy, in which reconstruction of RGEA and RGEV and cervical anastomosis using gastric conduit were performed simultaneously: (1) Immediate reconstruction of the RGEA and RGEV and long-term maintenance of the blood flow effectively. (2) Effective tension reduction of gastric conduit anastomosis and vascular anastomosis.

The short-term ischemia reperfusion process can help the gastric conduit adapt to the transient hypoxia environment, which may lower the risk of postoperative anastomotic leakage (4). However, the venous injury may cause severe gastric conduit congestion and even microcirculation thrombosis, which can seriously affect the healing of the anastomosis and impede the blood reperfusion after vascular reconstruction, resulting in postoperative anastomotic fistula possibly. Therefore, we recommend a vein-first principle of vascular anastomosis.

In terms of the anticoagulation therapy after vascular construction, we suggest that anticoagulation therapy should be administered intraoperatively as no previous reports available. Since the average inner diameter of the proximal end of RGEA is similar to that of the coronary artery, we referred to the anticoagulation guideline for coronary artery bypass graft surgery (5).

There are several inherent limitations in the present study. First, the number of patients who underwent vascular reconstruction and cervical anastomosis in McKeown esophagectomy was limited. Second, whether RGEA reconstruction alone could be an alternative to both RGEA and RGEV reconstruction should arouse more attention.

It is recommended to reconstruct the RGEA and RGEV immediately in the vein-first order, after which the simultaneous cervical anastomosis is feasible and reliable in McKeown esophagectomy.

## Data Availability Statement

The original contributions presented in the study are included in the article/Supplementary Material, further inquiries can be directed to the corresponding author/s.

## Ethics Statement

The studies involving human participants were reviewed and approved by The ethics committee of the Second Affiliated Hospital of Soochow University. The patients/participants provided their written informed consent to participate in this study.

## Author Contributions

LC, JZ, and DC: manuscript writing/editing and data analysis. LC and JZ: data collection. YS and WY: protocol/project development and manuscript writing/editing. All authors contributed to the article and approved the submitted version.

## Conflict of Interest

The authors declare that the research was conducted in the absence of any commercial or financial relationships that could be construed as a potential conflict of interest.

## Abbreviations

RGEA: right gastroepiploic artery
RGEV: right gastroepiploic vein.
