# Supplementary material for: Simultaneous Vascular Reconstruction and Cervical Anastomosis in McKeown Esophagectomy
Source: Front Surg. 2021 Apr 8;8:646811. doi: 10.3389/fsurg.2021.646811 (PMC8060638; doi:10.3389/fsurg.2021.646811)
Supplement: Supplementary file 1 [file Table_1.DOCX]

**Supplemental Table 1.** Anticoagulation therapy for patients who underwent vascular reconstruction.

|  | Anticoagulant | Dose | Methods | Treatment period |
| --- | --- | --- | --- | --- |
| Intraoperative | diluted heparin | 5000U/single dose | injected into the distal artery | instant application |
| Postoperative | low molecular weight heparin | 0.4ml/qd | subcutaneously injected | before removing the drainage tube |
| Long-term | aspirin | 100mg/qd | oral application | 1 year after removing the drainage tube |
